# BLAT2DOLite: An Online System for Identifying Significant Relationships between Genetic Sequences and Diseases

**DOI:** 10.1371/journal.pone.0157274

**Published:** 2016-06-17

**Authors:** Liang Cheng, Shuo Zhang, Yang Hu

**Affiliations:** 1 College of Bioinformatics Science and Technology, Harbin Medical University, Harbin 150081, PR China; 2 School of Management, Harbin University of Commerce, Harbin 150028, PR China; 3 School of Life Science and Technology, Harbin Institute of Technology, Harbin 150001, PR China; Tianjin University, CHINA

## Abstract

The significantly related diseases of sequences could play an important role in understanding the functions of these sequences. In this paper, we introduced BLAT2DOLite, an online system for annotating human genes and diseases and identifying the significant relationships between sequences and diseases. Currently, BLAT2DOLite integrates Entrez Gene database and Disease Ontology Lite (DOLite), which contain loci of gene and relationships between genes and diseases. It utilizes hypergeometric test to calculate P-values between genes and diseases of DOLite. The system can be accessed from: http://123.59.132.21:8080/BLAT2DOLite. The corresponding web service is described in: http://123.59.132.21:8080/BLAT2DOLite/BLAT2DOLiteIDMappingPort?wsdl.

## Introduction

Identifying significantly related diseases of genes has drawn more and more attention in interpreting molecular functions [1–13]. For example, through exploiting the significant relationships between diseases and altered genes by promyelocytic leukemia protein (PML) based on microarray analysis, Anida et al. identified the role of PML in diseases other than cancers [1]. Jiny et al. exploited overlapping between disease-related genes and inflammatory genes to explore core transcriptional regulators of inflammatory genes in coronary artery disease [2].

Enrichment analysis is an effective method to identify the significant relationships between diseases and genes. To this end, a disease vocabulary and a data set of associations between diseases and genes are needed first. Many databases are suitable for this purpose, in which Online Mendelian Inheritance in Man (OMIM) [14] and Gene References Into Function (GeneRIF) [15] have been most commonly used. OMIM is a database that concerns genetic disorders and its induced genes. In contrast, GeneRIF is more comprehensive, which is initiated by the National Library of Medicine (NLM) to link published data to Entrez Gene entries. GeneRIF consists of an Entrez Gene ID, a short text (under 255 characters), and the PubMed identifier (PMID) of the publication that provides evidence for the assertion in that text. Then, gene-disease relationships from the GeneRIF database were discovered [16] by Unified Medical Language System (UMLS) [17] MetaMap Transfer tool (MMTx) [18]. Here, disease terms were filtered by Disease Ontology (DO) [19]. In consideration that a simplified version of vocabulary could be helpful for integrating overview of molecular and cellular biology by combining and removing fine-grained terms [20,21], a simplified vocabulary list from the DO called Disease Ontology Lite (DOLite) [22] was constructed for enrichment analysis.

Many tools have been developed for the ease of accessing the significant relationships between diseases and genes, such as DAVID [23], FunDO [22], DOSE [24], DOSim [25], and GeneAnswer [26]. DAVID was an early bioinformatics analytic tool for systematically extracting biological meaning from large gene/protein lists. In contract, FunDO, DOSE, DOSim, and GeneAnswer can be used to study the significant relationship between diseases and genes. Though gene symbols or gene IDs can be analysed by existing tools, sequence data cannot be processed by all of these five tools. With the development of the next-generation sequencing technology, a large number of sequence data have been produced. Meanwhile, sequence alignment tools have been developed to identify the loci of sequence [27,28]. Therefore, analysing the relationship between sequence data and diseases is a critical challenge.

In this paper, we presented an online tool BALT2DOLite to annotate human genes and diseases, and to identify the significantly related diseases of sequences. Through BLAT2DOLite, sequences were first mapped to their locus by BLAT, and then these sequences were mapped to genes. According to associations between diseases of DOLite and genes, hypergeometric test was exploited to calculate the significant relationships between them. The system can be accessed from: http://123.59.132.21:8080/BLAT2DOLite. For easing to invoke the functions of BLAT2DOLite locally, a web service was also provided, which is described in: http://123.59.132.21:8080/BLAT2DOLite/BLAT2DOLiteIDMappingPort?wsdl.

## Materials and Methods

### Data Collection

Data sets of BLAT2DOLite were from open source databases. All of these databases were listed in the Table 1. For example, disease terms and relationships between these diseases and genes were from DOLite [22]. Currently, DOLite contains 15,016 associations between 560 diseases and 3,966 genes. In addition, a human reference genome (hg19) [29] was originated from UCSC Genome Browser [30]. In order to retrieve mappings from locus to genes, Entrez Gene database [31] was integrated in our system.

**Table 1:** Data sources and tools used for identifying significant relationships between sequences and diseases.

Data source / tool	Web site (Date of download)
DO	https://diseaseontology.svn.sourceforge.net/svnroot/diseaseontology/trunk/ (Jan 2016)
DOLite	http://fundo.nubic.northwestern.edu/ (Jan 2016)
Entrez Gene database	http://www.ncbi.nlm.nih.gov/gene (Jan 2016)
hg19	ftp://hgdownload.cse.ucsc.edu/gbdb/hg19/ (Jan 2016)
BLAT	http://genome.ucsc.edu/cgi-bin/hgBlat (Jan 2016)

### The Process of BLAT2DOLite

**Fig 1:**
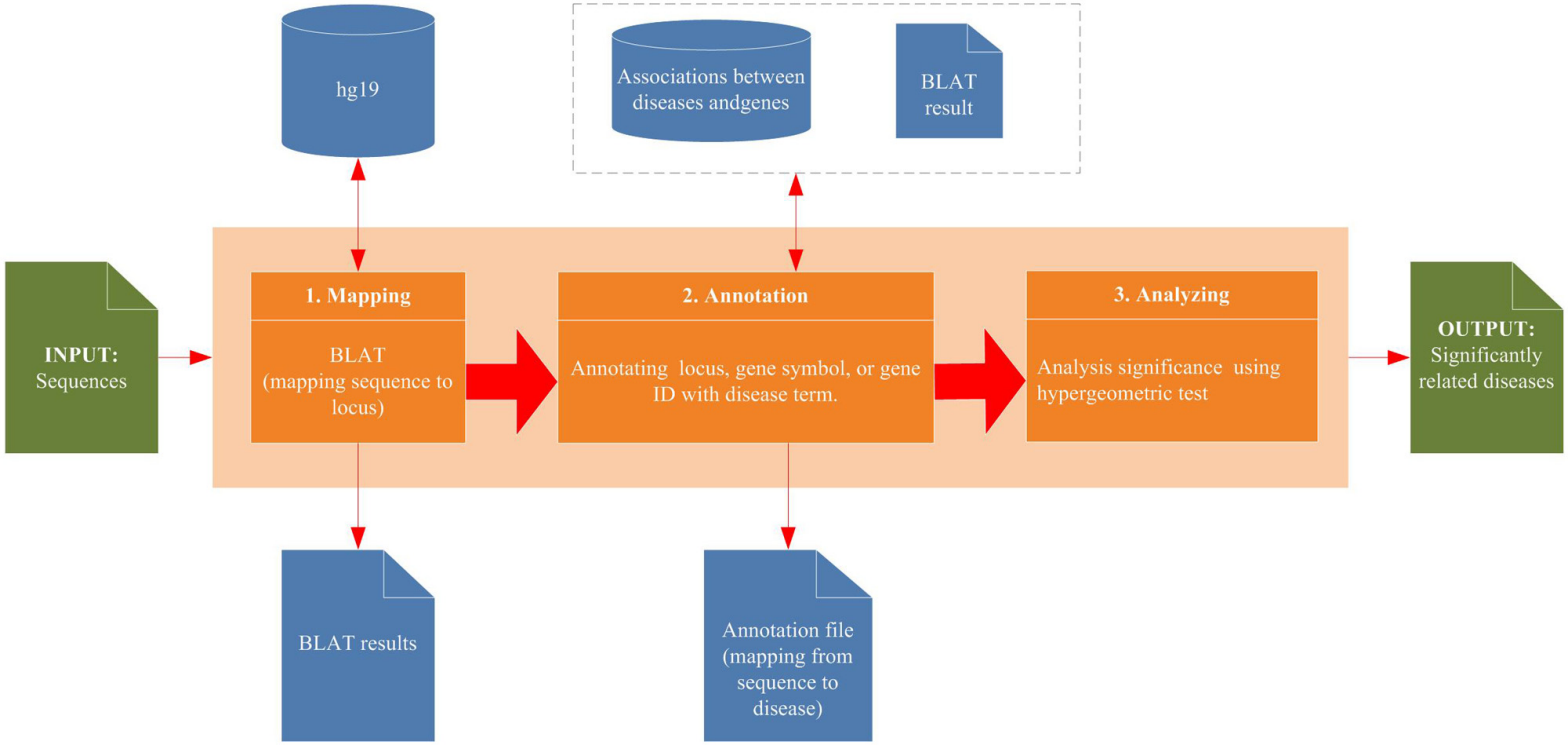
The process of BLAT2DOLite.

According to our system, significantly related diseases of sequences could be identified, the process of which was described in the Fig 1 as following.

#### Step 1: Mapping sequence to locus

Sequences could be mapped to a human reference genome (hg19) by BLAT, which is an open source software for finding loci of sequences. After mapping by BLAT [32], the location with the longest sequence mapping is selected.

#### Step 2: Annotating locus, gene symbol, or gene ID with diseases

Sequences in the previous step could be related to genes based on their locus. Here, two types of relevance were used for annotation: 1) Contain: the loci of gene is in the locus of sequences or the locus of sequences is in the loci of gene; 2) Intersect: The loci of gene covers the locus of sequences partly. Then, based on the relationships between genes and diseases of DOLite, sequences could be annotated with human diseases.

#### Method for analyzing the significant relationship between sequences and diseases

Here, hypergeometric test was utilized for analyzing the significant relationship between sequences and diseases. The formula for calculating P-value is as follows:

$$p-value=1-\underset{0\leq i\leq x}{\sum }\frac{C_{M}^{i}\times C_{N-M}^{k-i}}{C_{N}^{k}}.$$ (1)

Taking breast cancer as an example, N indicates the number of genes related by all of diseases, M indicates the number of genes related with breast cancer, k indicates the number of genes related with sequences, x indicates the number of common genes related with sequences and breast cancer.

### Implementation

**Fig 2:**
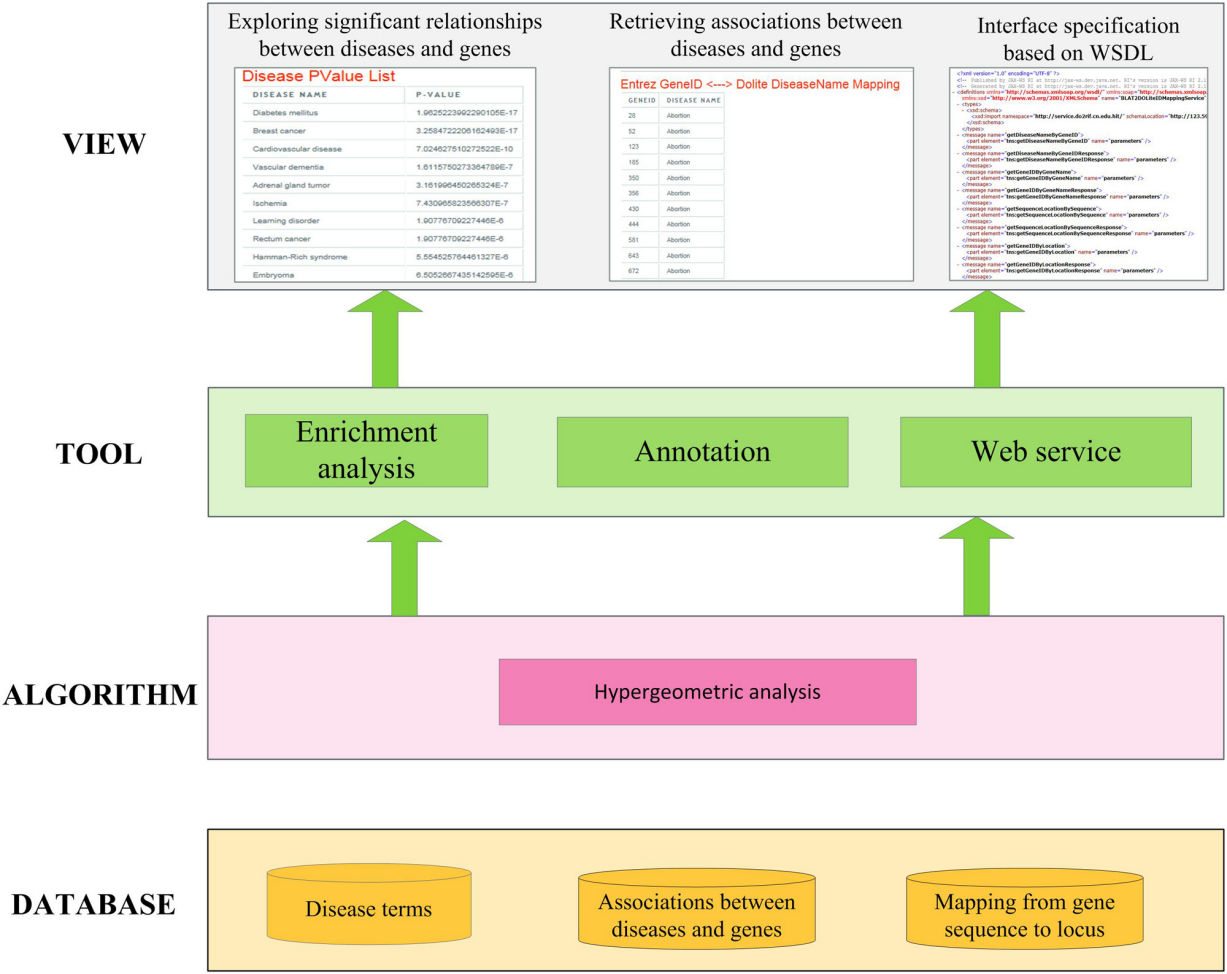
System overview of BLAT2DOLite.

BLAT2DOLite has been implemented on a JavaEE framework and run on the web server (2-core (2.26 GHz) processors) of UCloud [33]. The four-layer architecture involving DATABASE, ALGORITHM, TOOLS, and VIEW layer is shown in the Fig 2. The detailed description of the architecture is as following.

1. DATABASE layer. This layer stores locus of genes, disease terms and associations between human genes and diseases. These data are used by ALGORITHM layer and TOOL layer for annotating human genes and diseases and identifying the significant relationships between human diseases and sequences, respectively.
2. ALGORITHM layer. Hypergeometric analysis is implemented for calculating the significant relationships between diseases and sequences.
3. TOOL layer. The system provides two types of functions including annotating human genes and diseases and identifying the significant relationships between sequences and diseases. Furthermore, the functions of this system can be accessed based on our web service [34].
4. VIEW layer. Webpages are provided for viewing all the results based on TOOL layer. For example, the relationship between human diseases and genes can be shown, and the significant relationship between sequences and diseases can also be obtained. In addition, the interface specification of our web service can be accessed from the web.

## Results

The system could be used for annotating human genes and diseases, and identifying the significant relationships between sequences and diseases. The details about the access to these two functions are described as follows.

### A case for annotating human genes and diseases

**Fig 3:**
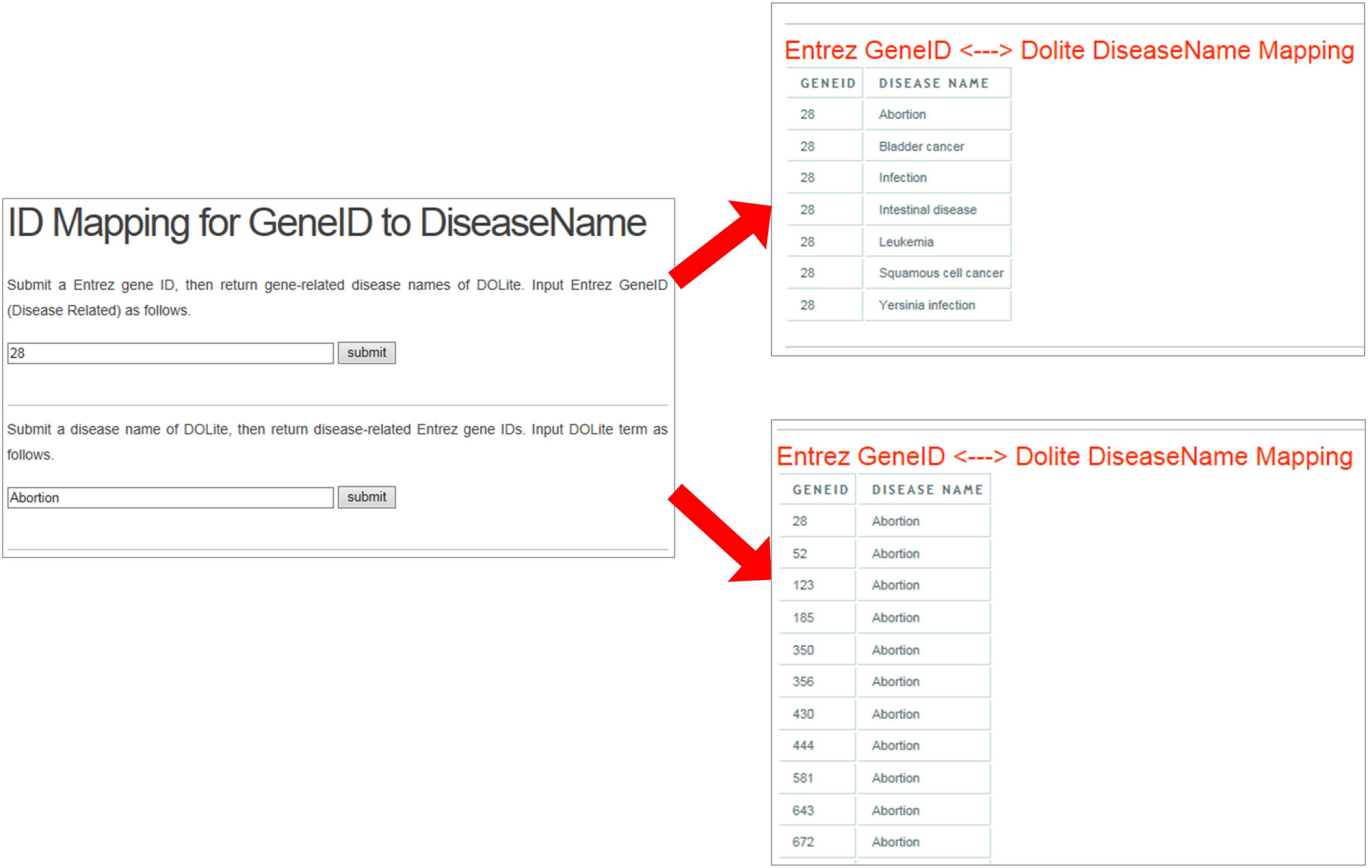
Schematic workflow of annotating human genes and diseases.

Human genes and diseases can be annotated from the web (http://123.59.132.21:8080/BLAT2DOLite/geneid2diseasename.jsp), a case of which is shown in Fig 3.

According to the figure, the system could return diseases after submitting an Entrez Gene ID. In this case, the inputted gene ID was ‘28’. And diseases could be affected by this gene were listed in the result page, such as bladder cancer, squamous cell cancer, and so on. Similarly, the system could return Entrez Gene IDs after submitting a disease term. In this case, the inputted disease term was ‘Abortion’. And gene IDs could induce this disease were listed in the result page, such as ‘52’, ‘153, and so on.

### A case for identifying the significant relationships between sequences and diseases

**Fig 4:**
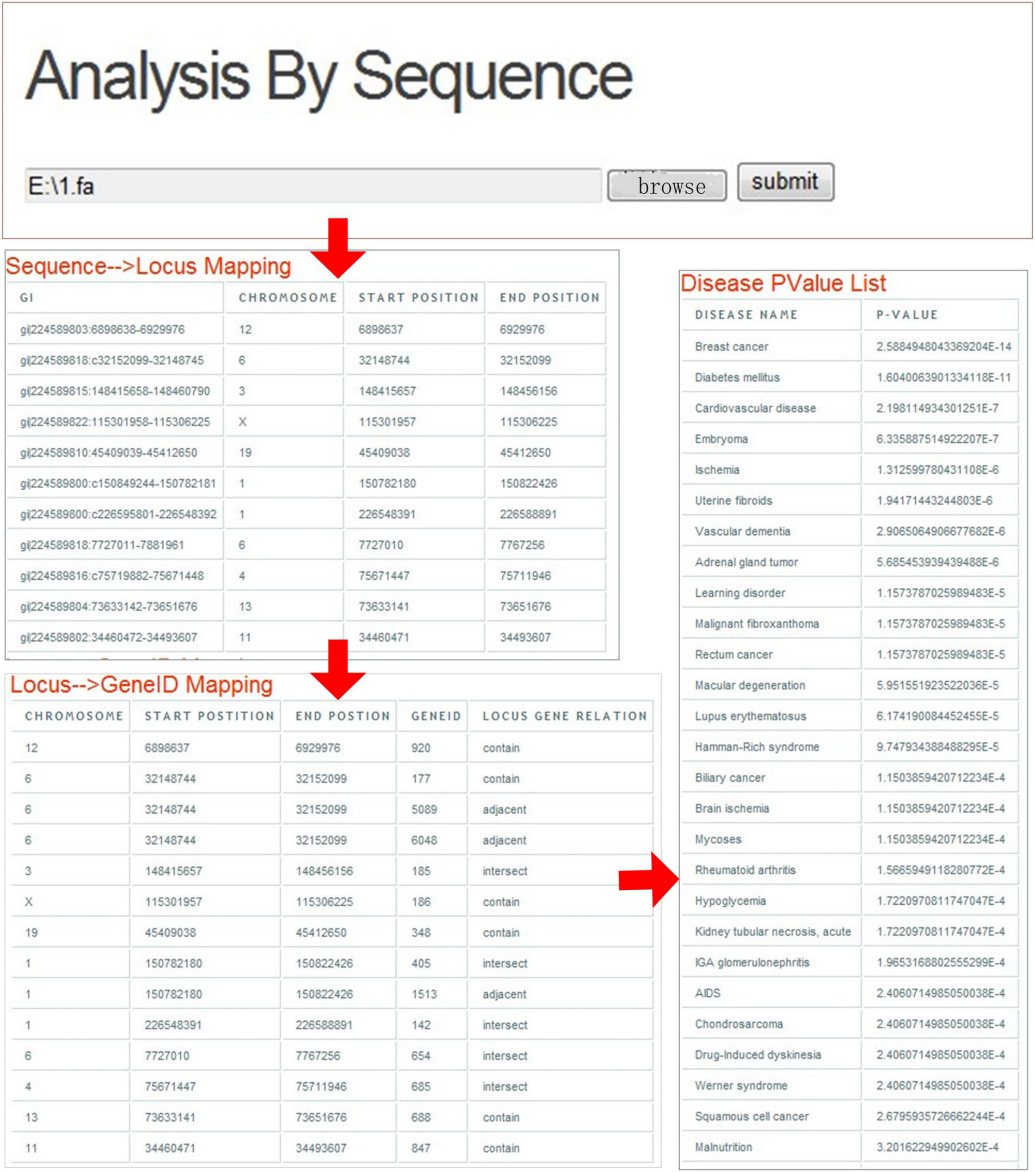
Schematic workflow of identifying significant relationships between sequences and diseases.

The significantly related diseases of sequences could be identified from the web (http://123.59.132.21:8080/BLAT2DOLite/sequence.jsp), a case of which is shown in Fig 4.

In this system, DNA sequences with FASTA format, in which nucleotides are represented using single-letter codes, could be submitted as an input. This format originates from the FASTA software package [35], but has now become a standard in the field of bioinformatics.

According to the schematic workflow of BLAT2DOLite in the Fig 1. First, sequences could be mapped to locus in the hg19. This mapping result could be returned to the result page. Next, the locus of these sequences could be mapped to Entrez Gene IDs based on the integrated Entrez Gene database. The corresponding associations between locus of these sequences and the locus of genes could also be shown in the result page. Then, these mapped gene IDs were annotated with diseases by BLAT2DOLite. The annotation result was not shown in this result page, in case the annotation function was provided by the system in the annotation page. Finally, the hypergeometric test was used to calculate P-values between these mapped genes and each disease of DOLite. Diseases with P-value less than 0.05 could be shown in the result page.

In the case shown in the Fig 4, the sequences in the web page were used as input. And the result page including ‘Sequence-Locus Mapping’, ‘Locus-Gene ID Mapping’ and ‘Disease P-value’ sections could be returned. In the ‘Sequence-Locus Mapping’ section, the identifiers of mapped sequences were shown in the first column of the table. And the mapped chromosome, start position, and end position of sequences in the same line were listed in the next three columns, respectively. For example, sequences gi|224589803:6898638–6929976 were mapped to locus from 6898637 to 6929976 in the twelfth chromosome. In the ‘Locus-Gene ID Mapping’ section, the relationships between loci of sequences and Entrez Gene IDs could be obtained. For example, in the first line of the result table of this section, the loci of gi|224589803:6898638–6929976 was mapped to Entrez Gene ‘920’. In the ‘Disease P-value’ section, significantly related diseases of these sequences were listed ranked by the P-values in descending order. In this case, diabetes mellitus was identified as the most significant disease of these sequences, so it was listed in the top of the corresponding result table.

### Web service of BLAT2DOLite

All the functions of our system were implemented as a web service through the JAVA API for XML Web Services (JAX-WS). The detailed description of our web service can be accessed from the following website: http://123.59.132.21:8080/BLAT2DOLite/BLAT2DOLiteIDMappingPort?wsdl. According to the interface of our web service, users can easily introduce the function of BLAT2DOLite locally.

## Conclusion

In this paper, an online system was presented for annotating human genes and diseases and identifying the significant relationships between sequences and diseases. For identifying the relationships between sequences and diseases, BLAT and the Entrez Gene database were integrated to map sequence to Entrez Gene ID. In this system, associations between human genes and diseases of DOLite were utilized for calculating the significant relationships between them. Furthermore, a web service was provided for the ease of introducing the function of BLAT2DOLite locally.

## References

[1] Sarajlić A, Janjić V, Stojković N, Radak D, Pržulj N (2013) Network topology reveals key cardiovascular disease genes. PloS one 8: e71537 10.1371/journal.pone.0071537 23977067 PMC3744556

[2] Nair J, Ghatge M, Kakkar VV, Shanker J (2014) Network analysis of inflammatory genes and their transcriptional regulators in coronary artery disease. PloS one 9: e94328 10.1371/journal.pone.0094328 24736319 PMC3988072

[3] Cheng X, Kao H-Y (2012) Microarray analysis revealing common and distinct functions of promyelocytic leukemia protein (PML) and tumor necrosis factor alpha (TNF α) signaling in endothelial cells. BMC genomics 13: 1. 22947142 10.1186/1471-2164-13-453 PMC3542097

[4] Xiang Y, Payne PR, Huang K (2012) Transactional database transformation and its application in prioritizing human disease genes. IEEE/ACM Transactions on Computational Biology and Bioinformatics (TCBB) 9: 294–304. 10.1109/TCBB.2011.58 PMC4047992 21422495

[5] Shashni B, Sakharkar KR, Nagasaki Y, Sakharkar MK (2013) Glycolytic enzymes PGK1 and PKM2 as novel transcriptional targets of PPARγ in breast cancer pathophysiology. Journal of drug targeting 21: 161–174. 10.3109/1061186X.2012.736998 23130662

[6] Danilov A, Shaposhnikov M, Plyusnina E, Kogan V, Fedichev P, Moskalev A. (2013) Selective anticancer agents suppress aging in Drosophila. Oncotarget 4: 1507–1526. 24096697 10.18632/oncotarget.1272 PMC3824538

[7] Janjić V, Pržulj N (2012) The core diseasome. Molecular Biosystems 8: 2614–2625. 10.1039/c2mb25230a 22820726

[8] Sullivan J, Karra K, Moxon SA, Vallejos A, Motenko H, Wong J, et al. (2013) InterMOD: integrated data and tools for the unification of model organism research. Scientific reports 3. 10.1038/srep01802 PMC3647165 23652793

[9] Zhao M, Sun J, Zhao Z (2013) TSGene: a web resource for tumor suppressor genes. Nucleic acids research 41: D970–D976. 10.1093/nar/gks937 23066107 PMC3531050

[10] M Vazquez-Naya J, Martinez-Romero M, B Porto-Pazos A, Novoa F, Valladares-Ayerbes M, Pereira J, et al (2010) Ontologies of drug discovery and design for neurology, cardiology and oncology. Current pharmaceutical design 16: 2724–2736. 20642429 10.2174/138161210792389199

[11] Liu Y, Zeng X, He Z, Zou Q (2016) Inferring microRNA-disease associations by random walk on a heterogeneous network with multiple data sources. IEEE/ACM Trans Comput Biol Bioinform. 10.1109/TCBB.2016.2550432 27076459

[12] Zou Q, Li J, Hong Q, Lin Z, Wu Y, Shi H, et al (2015) Prediction of microRNA-disease associations based on social network analysis methods. BioMed research international 2015: 810514 10.1155/2015/810514 26273645 PMC4529919

[13] ZENG X, LIAO Y, Zou Q (2016) Prediction and validation of disease genes using HeteSim Scores. 10.1109/TCBB.2016.2520947 26890920

[14] Amberger JS, Bocchini CA, Schiettecatte F, Scott AF, Hamosh A (2015) OMIM. org: Online Mendelian Inheritance in Man (OMIM^®^), an online catalog of human genes and genetic disorders. Nucleic acids research 43: D789–D798. 10.1093/nar/gku1205 25428349 PMC4383985

[15] Lu Z, Cohen KB, Hunter L. GeneRIF quality assurance as summary revision; 2007 NIH Public Access pp. 269. 10.1142/9789812772435_0026 PMC2652871 17990498

[16] Osborne JD, Flatow J, Holko M, Lin SM, Kibbe WA, Zhu LJ, et al (2009) Annotating the human genome with Disease Ontology. BMC genomics 10: S6. 10.1186/1471-2164-10-S1-S6 PMC2709267 19594883

[17] Lindberg DA, Humphreys BL, McCray AT (1993) The Unified Medical Language System. Methods of information in medicine 32: 281–291. 8412823 10.1055/s-0038-1634945 PMC6693515

[18] Meystre S, Haug PJ (2005) Evaluation of medical problem extraction from electronic clinical documents using MetaMap Transfer (MMTx). Studies in health technology and informatics 116: 823–828. 16160360

[19] Schriml LM, Arze C, Nadendla S, Chang Y-WW, Mazaitis M, Felix V, et al (2012) Disease Ontology: a backbone for disease semantic integration. Nucleic acids research 40: D940–D946. 10.1093/nar/gkr972 22080554 PMC3245088

[20] Adams MD, Celniker SE, Holt RA, Evans CA, Gocayne JD, Amanatides PG, et al (2000) The genome sequence of Drosophila melanogaster. Science 287: 2185–2195. 10731132 10.1126/science.287.5461.2185

[21] Shah N, Fedoroff NV (2004) CLENCH: a program for calculating Cluster ENriCHment using the Gene Ontology. Bioinformatics 20: 1196–1197. 14764555 10.1093/bioinformatics/bth056

[22] Du P, Feng G, Flatow J, Song J, Holko M, Kibbe WA, et al (2009) From disease ontology to disease-ontology lite: statistical methods to adapt a general-purpose ontology for the test of gene-ontology associations. Bioinformatics 25: i63–68. 10.1093/bioinformatics/btp193 19478018 PMC2687947

[23] Huang DW, Sherman BT, Lempicki RA (2009) Systematic and integrative analysis of large gene lists using DAVID bioinformatics resources. Nature protocols 4: 44–57. 10.1038/nprot.2008.211 19131956

[24] Yu G, Wang L-G, Yan G-R, He Q-Y (2015) DOSE: an R/Bioconductor package for disease ontology semantic and enrichment analysis. Bioinformatics 31: 608–609. 10.1093/bioinformatics/btu684 25677125

[25] Li J, Gong B, Chen X, Liu T, Wu C, Zhang F, et al (2011) DOSim: An R package for similarity between diseases based on Disease Ontology. BMC bioinformatics 12: 1. 21714896 10.1186/1471-2105-12-266 PMC3150296

[26] Feng G, Shaw P, Rosen ST, Lin SM, Kibbe WA (2012) Using the bioconductor GeneAnswers package to interpret gene lists. Next Generation Microarray Bioinformatics: Methods and Protocols: 101–112. 10.1007/978-1-61779-400-1_7 22130876

[27] McGinnis S, Madden TL (2004) BLAST: at the core of a powerful and diverse set of sequence analysis tools. Nucleic acids research 32: W20–W25. 15215342 10.1093/nar/gkh435 PMC441573

[28] Zou Q, Hu Q, Guo M, Wang G (2015) HAlign: Fast multiple similar DNA/RNA sequence alignment based on the centre star strategy. Bioinformatics 31: 2475–2481. 10.1093/bioinformatics/btv177 25812743

[29] Bioinformatics UG (2011) GRCh37/hg19 assembly.

[30] Meyer LR, Zweig AS, Hinrichs AS, Karolchik D, Kuhn RM, Wong M, et al (2013) The UCSC Genome Browser database: extensions and updates 2013. Nucleic Acids Res 41: D64–69. 10.1093/nar/gks1048 23155063 PMC3531082

[31] Maglott D, Ostell J, Pruitt KD, Tatusova T (2011) Entrez Gene: gene-centered information at NCBI. Nucleic acids research 39: D52–D57. 10.1093/nar/gkq1237 21115458 PMC3013746

[32] Kent WJ (2002) BLAT—the BLAST-like alignment tool. Genome research 12: 656–664. 11932250 10.1101/gr.229202 PMC187518

[33] Sqalli MH, Al-Saeedi M, Binbeshr F, Siddiqui M. UCloud: A simulated Hybrid Cloud for a university environment; 2012 IEEE pp. 170–172.

[34] Vaughan-Nichols SJ (2002) Web services: Beyond the hype. Computer: 18–21.

[35] Pearson WR, Lipman DJ (1988) Improved tools for biological sequence comparison. Proc Natl Acad Sci U S A 85: 2444–2448. 3162770 10.1073/pnas.85.8.2444 PMC280013

